# Vitamin A and D in allergy: from experimental animal models and cellular studies to human disease

**DOI:** 10.1007/s40629-018-0054-2

**Published:** 2018-02-20

**Authors:** Karin Hufnagl, Erika Jensen-Jarolim

**Affiliations:** 1 The Interuniversity Messerli Research Institute of the University of Veterinary Medicine Vienna, Medical University Vienna and University Vienna, Vienna, Austria; 2 Institute of Pathophysiology and Allergy Research, Center for Pathophysiology, Infectiology and Immunology, Medical University Vienna, Währinger Gürtel 18–20, 1090 Vienna, Austria

**Keywords:** Retinoic acid, Vitamin D3, Allergenicity, Mouse model, Immunomodulation

## Abstract

**Introduction:**

Vitamins A and D are able to modulate innate and adaptive immune responses and may therefore influence the development and the course of allergic diseases.

**Materials and methods:**

This article reviews the current evidence for the experimental effects of vitamins A and D *in vivo* in animal models and on immune cells *in vitro*, and discusses their translational implication. A systematic literature search over the last 10 years was performed using MEDLINE and PubMed databases.

**Results:**

Deficiencies of vitamin A or vitamin D in mouse models of allergic asthma seem to exacerbate allergic symptoms along with enhanced lung inflammation and Th2 cytokine production. In contrast, supplementation regimes especially with vitamin D were able to attenuate symptoms in therapeutic mouse models. The active metabolites retinoic acid (RA) and 1,25-dihydroxyvitamin D3 (VD3) induced tolerogenic dendritic cells (DCs) and up-regulated T‑regulatory cells in the allergic sensitization phase, which likely contributes to tolerance induction. Additionally, RA and VD3 maintained the stability of eosinophils and mast cells in the effector phase, thereby reducing allergic mediator release. Thus, both active vitamin metabolites RA and VD3 are able to influence allergic immune responses at several immunological sites.

**Conclusion:**

Animal studies predict that vitamin A and D may also be attractive players in the control of allergy in humans. Whether these experimental observations can be translated to the human situation remains open, as results from clinical trials are controversial.

## Introduction

Vitamins and their metabolites regulate tissue growth, differentiation and embryonic development and have an important control function in the immune homeostasis by influencing both innate and adaptive immune responses. Vitamins A and D are distinct from other vitamins as their metabolites, retinoic acid (RA) and 1,25-dihydroxyvitamin D3 (VD3), are synthesized from precursors by different body tissues and they exert their effects on target cells by binding to nuclear hormone receptors.

### Vitamin A

is essential for pre- and postnatal development, eyesight and reproduction, but it also has an important role in the maintenance of the immune system [1]. Vitamin A is taken up in form of retinol or retinyl esters from animal-derived food sources (e. g. fish, liver, milk, eggs) or in form of precursors such as carotenoids from plant food (e. g. carrots, red peppers, lettuce). Vitamin A is stored mainly in the liver. In liver cells, retinol is oxidized to retinal by alcohol dehydrogenases and metabolized to its main active metabolite RA [2]. In the target tissue, the two isoforms all-trans-RA and 9‑cis-RA bind to nuclear retinoic acid receptors [3] and/or retinoid X receptors (RXR), regulating over 500 retinoid-responsive genes [2, 4]. Formation of RA is catalysed by enzymes called retinal dehydrogenases (RALDH), which are expressed in epithelial cells and in immune cells such as macrophages and dendritic cells (DCs) [1]. Recent work has revealed that these enzymes are present not only in gut-associated cells but also in the lung, which has prominent extrahepatic stores of vitamin A [3].

### Vitamin D

promotes calcium absorption in the gut and helps maintain calcium and phosphate levels to promote bone health [5]. Apart from having neuromuscular and cardiovascular effects it is known to regulate immune cell functions [6]. Vitamin D is acquired via the diet (especially contained in fatty fish) and large amounts are synthesized in the skin from 7‑dehydrocholesterol after exposure to UVB light. After hydroxylation in the liver, the resulting 25-hydroxyvitamin D3 is further hydroxylated in the kidneys by the enzyme 1‑α-hydroxylase resulting in VD3, which is the physiologically active metabolite. VD3 in the target tissue binds to the nuclear vitamin D receptor (VDR), which heterodimerizes with nuclear receptors of the retinoic X receptor family, and binds to VD3 response elements in the promoters of VD3-responsive genes [6]. Both rate-limiting enzymes 1‑α-hydroxylase and the vitamin D receptor are expressed in many tissues, including the lungs, colon, skin, lymph nodes and cells of the immune system. Macrophages, DCs and T and B cells are therefore able to produce VD3 locally, which can act on immune cells in an autocrine or paracrine manner by binding to the vitamin D receptor [7, 8].

## The immunomodulatory role of vitamin A/RA and VD3

The immunomodulatory potential of RA and VD3 was investigated since long [9] and recently their general influence on immune cells was reviewed in detail [1, 6]. Here we review reports on the innate and adaptive immune cells which are key players in allergic diseases.

### Vitamin A/RA

#### B cells

The major influence on RA signalling in B cells concerns immunoglobulin class switching [1]. RA from intestinal DCs promotes generation of IgA+ producing B cells and potentiates memory B cell differentiation [1]. Consequently, vitamin A deficiency leads to a severe decrease in intestinal and serum IgA levels [10].

#### APCs, T cells/Treg cells

On the T cellular level RA influences the homing of both CD4+ and CD8+ T cells by enhancing the expression of the gut-homing molecules α4ß7 and CCR9, promoting preferential migration to the gut-associated lymphoid tissue (GALT) [11]. Most importantly, local production of RA by mucosal DCs together with TGF-ß can induce naive T cells to differentiate into FOXP3+ regulatory T cells (Tregs), thereby maintaining immune tolerance [12]. This phenomenon is not restricted to the gut-associated lymphoid tissue. Alveolar macrophages in the lung were found to promote the induction of FOXP3+ Tregs, which then again mediate respiratory tolerance through the production of RA and TGF-ß [13]. A reciprocal regulatory effect of RA is seen in the suppression of Th17 and Th1 cell differentiation [14]. While under steady-state conditions RA maintains tolerance at mucosal surfaces inducing tolerogenic DCs and Tregs, RA can also induce inflammatory DCs and differentially affect T cell responses in already polarized T cells during infection or autoimmunity [1, 14].

#### ILCs

In innate lymphocytes RA promotes the generation of ILC3s and antagonizes ILC2 function, highlighting the multiple functions of RA in immune cells [15].

### Vitamin D/VD3

#### APCs

VD3 has a stimulatory effect on monocytes and macrophages, enhancing chemotaxis and activating the cathelicidin antimicrobial peptide [16]. VD3 can also inhibit the differentiation and maturation of DCs, leading to a tolerogenic state with lower antigen presentation but increased production of IL-10 [17]. As DCs express 1‑α-hydroxylase they can acquire high local concentrations of VD3. This notion is supported by studies in VDR/1-α-hydroxylase knockout mice, which show a large increase in mature DCs [17].

#### B cells

In B cells, which express 1‑α-hydroxylase and VDR upon activation, VD3 has an antiproliferative effect [18]. This effect is mediated by T helper cells but recent studies have shown that VD3 directly inhibits IgG secretion and the generation of memory and plasma cells, and promotes the apoptosis of B cells [8, 19].

#### T cells/Treg cells

VD3 inhibits the expression of the Th1 (IFN-y, TNF-α), Th9 (IL-9), Th17 (IL-17) and Th22 (IL-22) cytokines in T cells, but has been described to up-regulate Th2 (IL-4, IL-10) cytokines and to induce FOXP3+ regulatory T cells [8, 20]. A recent study on human CD4+ T cells showed a significant increase in the frequency of FOXP3+ regulatory T cells after stimulation with physiologically relevant levels of VD3 in combination with TGF-ß [21]. Interestingly, Th2 cell differentiation can also be inhibited by VD3 [20].

#### ILCs

In innate lymphoid cells VD3 seems to inhibit ILC2 activation and the expression of gut homing integrin [22].

Taken together, both active vitamin A and D metabolites seem to have a major influence on effector Th1/Th2/Th17 cell differentiation and regulatory T cell induction, which could also have implications for allergic disease development.

## Correlation of vitamin A/D deficiency with allergic immune responses *in vivo*

In recent decades animal models have significantly contributed to the understanding of pathophysiologic mechanisms of allergic diseases such as asthma, anaphylaxis or food allergy [23]. Here we will concentrate on animal models of vitamin deficiency or supplementation that operate with and reflect on vitamin A or vitamin D contents that are taken up via the diet.

In neonate mice vitamin A deficiency (VAD) resulted in impairment of oral tolerance induction [24] with insufficient Treg cell activation by DCs in mesenteric lymph nodes as possible underlying mechanism [25]. Interestingly, feeding of a single dose of vitamin A in adult mice could amplify the tolerogenic properties of mesenteric lymph node DCs [26]. Several studies reported that VAD in adult mice leads to increased airway hyperresponsiveness, lung inflammation and Th2 cytokine production ([27, 28]; Table 1). These mice were also prone to develop skin allergy upon oral antigen administration due to strong, IL-13 dependent IgE response [29]. Earlier observations on VAD found a dose-dependent alteration of asthma symptoms, with low-level VAD decreasing airway inflammation and hyperresponsiveness while high-level dietary vitamin A increased disease severity ([30, 31]; Table 1). The resulting hypothesis that excessive vitamin A intake in industrialized countries induces a Th2 bias and atopy prevalence is still very much under discussion [32].

**Table 1:** Effects of vitamin A and D in mouse models of airway inflammation associated with allergic asthma/rhinitis

Treatment protocol	Results	Reference
*Vitamin A/RA*		
Murine asthma model—ovalbumin (OVA) sensitization and challenge	Vitamin A deficiency exacerbates OVA-induced lung inflammation and type 2 cytokine production	[28]
Murine asthma model—OVA sensitization and challenge	Vitamin A deficiency decreases eosinophils, IL-4, IL-5 in lung together with hyperreactivity	[30]
Murine asthma model—OVA sensitization and challenge	RA administration leads to reduced airway inflammation by inhibiting Th2/Th17 differentiation	[46]
Murine house dust mice (HDM) induced allergic rhinitis	RA administration leads to reduced allergic rhinitis by inhibiting Th2 response and induction of Treg cells	[47]
*Vitamin D/VD3*		
HDM-induced airway hyperresponsiveness	Vitamin D deficiency results in increased airway eosinophilia and Th2 cells	[33]
Murine asthma model—OVA sensitization and challenge	Vitamin D deficiency is associated with airway hyperresponsiveness, high eosinophilia and proinflammatory cytokines in lungs of challenged mice	[34]
Murine asthma model—OVA sensitization and challenge	VD3 treatment is able to reduce chronic lung inflammation together with goblet cell hyperplasia and subepithelial collagen deposition	[48]
Murine asthma model—OVA sensitization and challenge	Transfer of VD3-treated CD8+ T cells is able to prevent airway hyperresponsiveness and inflammation in OVA-challenged recipient mice	[50]

*HDM* House dust mite, *OVA* Ovalbumin

Vitamin D deficiency in neonate mice seems to contribute to allergic disease severity with high eosinophilia and airway remodelling, which could be improved by vitamin D supplementation ([33]; Table 1). Similarly, supplementation of vitamin D reduced airway hyperresponsiveness and lung inflammation in mouse models of allergic asthma [34, 35]. In this respect, it was reported that eosinophils in vitamin D deficient mice seem to produce higher levels of mediator substances and are prone to spontaneous mediator release [36]. Effects could be demonstrated not only in asthma-related studies, but also in models of food allergy. In this experimental set-up vitamin D deficiency led to exacerbation of symptoms, possibly mediated by increased expression of IL-4 in mesenteric lymph nodes [37].

In allergen-specific immunotherapy (AIT), the only curative treatment for type I allergies, supplementation with 25-hydroxyvitamin D3 (inactive precursor of VD3) was able to enhance the beneficial effects in sensitized, vitamin D deficient mice [38]. This could have major implications for AIT in human allergic patients who often show a prevalence for vitamin D deficiency [39], especially in northern latitudes during wintertime which is exactly the time when pollen-specific AIT is initiated. Thus, a recent study demonstrated enhanced efficacy of sublingual AIT (SLIT) in combination with vitamin D supplementation in grass pollen allergic children [40].

## *In vivo* treatment with RA or VD3 as reference to underlying mechanisms of immunomodulation

There are a number of studies that focus on the *in vivo* administration of the major vitamin metabolites RA and VD3 and the effects of such treatment on immune cells of the innate as well as adaptive immune system.

Repeated antigen challenge in the presence of 9‑cis-RA reduced specific IgE responses and increased specific IgA responses in mice, probably via B cell derived IL-10 [41]. At the same time, it was reported that the IgE-repressive activity of RA was mediated mainly through the RA receptor alpha (RARα) by down-regulating IgE class-switching recombination [42]. Interestingly, by using lecithin:retinol acetyltransferase-deficient mice that are more susceptible to vitamin A deficiency [43] elevated serum levels of mouse mast cell protease-1 together with significantly reduced numbers of intestinal Treg cells were also found.

DCs at the borderline between the innate and adaptive immune system play a major role in tolerance induction and their phenotypic differentiation can be regulated by vitamin metabolites [44]. In this respect, DCs when differentiated under RA stimulation were able to prevent anaphylactic responses to oral peanut allergen challenge in mice [45]. The RA-generated DCs in this model displayed a tolerogenic mature phenotype expressing IL-10, TGF-ß and IL-27 [45]. Overall, *in vivo* administration of RA in murine models of allergic rhinitis or asthma was able to attenuate airway inflammation and hyperreactivity due to induction of FOXP3+ regulatory T cells and inhibition of Th2 and Th17 responses ([46, 47]; Table 1).

Concerning VD3, intraperitoneal administration at the time of airway antigen challenge led to reduced airway inflammation and remodeling in murine models of OVA-induced asthma ([48, 49]; Table 1). The protective role of VD3 in the lung of treated mice may be caused by inhibition of NF-kB activation, but also suppression of TGFß/SMAD signalling pathways was reported. Together with activation of the Nrf2/HO-1 pathway VD3 may be able to protect mice from antigen-induced oxidative injury and could be effective in the control of asthma [49].

Another recent report showed that VD3 could prevent the conversion of CD8+ T cells to IL-13 producing pathogenic effector cells in the lungs of antigen challenged mice ([50]; Table 1). The authors argue that vitamin D could therefore be beneficial for asthmatics, but restricted to a subpopulation of steroid-refractory asthmatics with increased numbers of CD8+IL-13+ T cells [50].

In mouse—and human—mast cells, key proinflammatory effector cells of allergic response, VD3 was able to inhibit mediator release in a vitamin D receptor-mediated manner [51]. These data are in line with a recent publication from Liu et al. [52]. They reported that VD3 is important to maintain the stability of mast cell by increasing vitamin D receptor expression together with inhibition of IgE-sensitized mast cell activation. In this respect, VD3 seems to interfere with two major key components of mast cell activation, the adaptor protein Myd88 and the FcεR1ß subunit of the high-affinity IgE receptor [52].

**Fig. 1:**
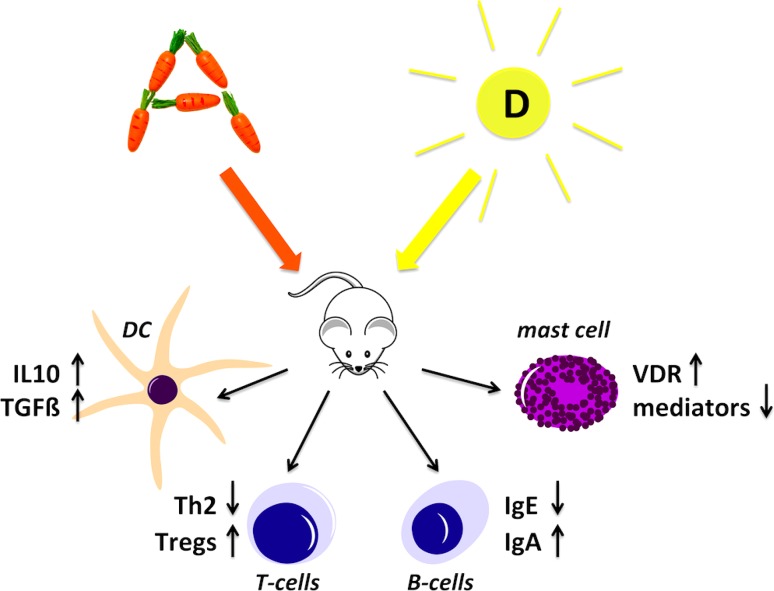
Vitamin A and vitamin D impact immune cells relevant in the control of allergy. In mouse models of allergic disease vitamin A and D, and their major metabolites retinoic acid (RA) and vitamin D 3 (VD3), down-regulate Th2 and IgE immune responses. Vitamin metabolite-treated dendritic cells (DCs) are prone to adapt a tolerogenic phenotype, leading to up-regulation of T‑regulatory cells (Tregs). Both vitamins also modulate T and B cell responses as well as mediator release from mast cells and eosinophils. (Copyrights from* ©motifolio.com, *and* Fotolia.com©Anastasia Anisenko*)

Thus, both active vitamin metabolites RA and VD3 are able to influence allergic immune responses at several immunological sites (Fig. 1).

## Can data from *in vivo* animal studies be correlated to the human situation?

The findings from *in vivo* animal studies suggest that vitamin A and D seem to have a major impact on immune cells as well as on the development, and in the case of vitamin D even on the treatment of allergic Th2-dominated diseases. The question remains whether these findings can be translated to the human situation, especially concerning the dosage of vitamin supplementation in connection with adequate vitamin status [6, 32]. Nonetheless, in some of the studies conducted in mice the doses of vitamins were comparable to those applied in humans [26, 38].

Studies in neonate mice predict that there could exist a “window of opportunity” for prevention of allergic diseases by vitamins A and D. This is reflected in human studies concentrating on the maternal vitamin A or D status that can be of importance for reducing the development of allergic diseases in children [53–55]. On the other hand, application of vitamin A as aerosol to asthmatic children was of no benefit [56], and no association between serum carotenoids and risk of asthma in young children could be established in a recently published longitudinal study [57]. Therefore, primary randomized controlled trials of the use of vitamin A to prevent or treat asthma that could elucidate the role of this vitamin in the human allergic disease are still missing.

Earlier studies reported that the vitamin D status in human allergic patients shows a nonlinear relationship with immune parameters relevant for allergic and asthmatic disease (e. g. serum IgE levels) [58]. Nevertheless, vitamin D supplementation trials in asthmatic and/or pollen allergic children gave positive results with regard to asthma exacerbations and improvement of immunotherapy [40, 59], similarly seen in case reports from adult asthmatics [60].

## Concluding remarks

The manifold impact of vitamin A and D on immune cell responses and profound evidence from animal studies provide hope that these molecules can help control allergy. More randomized controlled human studies are needed to underpin the potential of these vitamins and their metabolites in prevention or therapy of Th2-dominant allergic diseases including asthma. We propose that more emphasis should be put on the determination of the vitamin status of allergic patients and, especially for vitamin D, on prophylaxis in early life or during pregnancy.

## Abbreviations

AIT: Allergen-specific immunotherapy
APC: Antigen-presenting cell
DC: Dendritic cell
GALT: Gut-associated lymphoid tissue
HDM: House dust mite
IL: Interleukin
ILC: Innate lymphoid cell
OVA: Ovalbumin
RA: Retinoic acid
Treg: T-regulatory cell
VA: Vitamin A
VAD: Vitamin A deficiency
VDR: Vitamin D receptor
VD3: 1,25-dihydroxyvitamin D3
